# A Theoretical Model for Predicting Residual Stress Generation in Fabrication Process of Double-Ceramic-Layer Thermal Barrier Coating System

**DOI:** 10.1371/journal.pone.0169738

**Published:** 2017-01-19

**Authors:** Yan Song, Weijie Wu, Feng Xie, Yilun Liu, Tiejun Wang

**Affiliations:** 1 School of Chemical Engineering and Technology, Xi’an Jiaotong University, Xi’an, China; 2 State Key Laboratory for Strength and Vibration of Mechanical Structures, Xi’an Jiaotong University, Xi’an, China; 3 School of Aerospace Engineering, Xi’an Jiaotong University, Xi’an, China; Beihang University, CHINA

## Abstract

Residual stress arisen in fabrication process of Double-Ceramic-Layer Thermal Barrier Coating System (DCL-TBCs) has a significant effect on its quality and reliability. In this work, based on the practical fabrication process of DCL-TBCs and the force and moment equilibrium, a theoretical model was proposed at first to predict residual stress generation in its fabrication process, in which the temperature dependent material properties of DCL-TBCs were incorporated. Then, a Finite Element method (FEM) has been carried out to verify our theoretical model. Afterwards, some important geometric parameters for DCL-TBCs, such as the thickness ratio of stabilized Zirconia (YSZ, ZrO_2_-8%Y_2_O_3_) layer to Lanthanum Zirconate (LZ, La_2_Zr_2_O_7_) layer, which is adjustable in a wide range in the fabrication process, have a remarkable effect on its performance, therefore, the effect of this thickness ratio on residual stress generation in the fabrication process of DCL-TBCs has been systematically studied. In addition, some thermal spray treatment, such as the pre-heating treatment, its effect on residual stress generation has also been studied in this work. It is found that, the final residual stress mainly comes from the cooling down process in the fabrication of DCL-TBCs. Increasing the pre-heating temperature can obviously decrease the magnitude of residual stresses in LZ layer, YSZ layer and substrate. With the increase of the thickness ratio of YSZ layer to LZ layer, magnitudes of residual stresses arisen in LZ layer and YSZ layer will increase while residual stress in substrate will decrease.

## Introduction

Due to the excellent thermal isolation properties, thermal barrier coating system (TBCs), which is fabricated by Air Plasma Spray (APS) method, is being employed extensively in gas turbines and aircraft engines, to protect engine blades and other components from high temperature. Generally speaking, the structure of the as-sprayed TBCs consists of three different parts: the YSZ layer, consisting of ZrO_2_-8%Y_2_O_3_, was used as the top layer to provide thermal isolation; a nickel- or cobalt-based structural super-alloy substrate; and a thin NiCrA1Y coating was employed as bonding coating (BC) to coordinate thermal-mechanical properties between YSZ layer and substrate [1–4]. The demands for higher engine efficiency and performance, require the gas turbine to be operated under higher temperature. However, higher temperature usually leads to the occurrence of the phase transformations and sintering. This may not only shorten the life of the TBCs due to the formation of micro cracks, but also affect the fracture toughness of the YSZ layer. To overcome this contradiction, some new ceramic materials were proposed to overcome this requirement, such as Lanthanum Zirconate (LZ), Cerium Lanthanum Zirconate (LZ_7_C_3_) and LaMgAl_11_O_19_ (LMA), are proposed by their good thermal insulation properties and sintering resistance ability under high temperature, however, these new materials also have disadvantages, their low thermal expansion coefficients will cause large thermal mismatch, and their low fracture toughness will cause crack generation et al, these will have negative effect on durability of TBCs. Finally, it seems that the traditional TBCs, which has a single ceramic layer cannot meet all the demands of development of TBCs [5–8].

**Fig 1:**
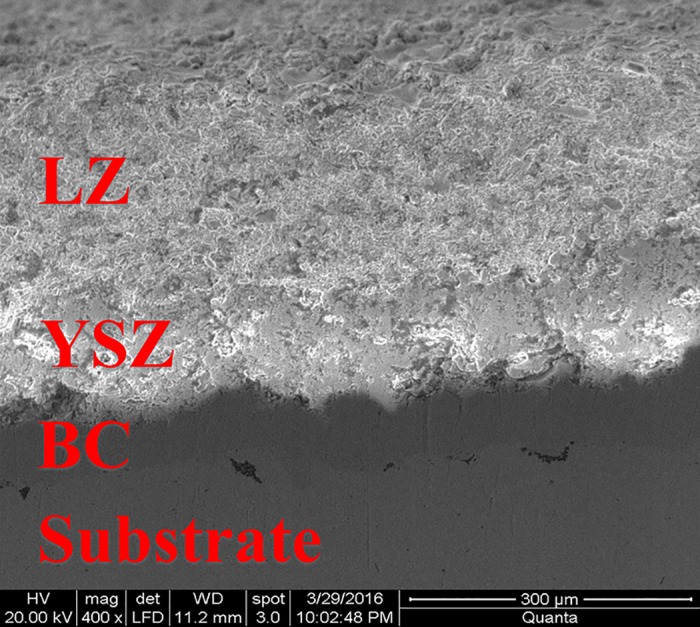
SEM photo of the cross section of a representative as-sprayed DCL-TBCs. Thicknesses of LZ layer, YSZ layer, BC and substrate are 220±20μm, 110±20μm, 65±20μm and 3±0.1mm, respectively. BC is fabricated by High-Velocity Oxygen-Fuel (HVOF) method, YSZ and LZ layers are fabricated by APS method.

For the reasons given above, the Double-Ceramic-Layers Thermal Barrier Coating system (DCL-TBCs) is proposed to overcome this problem. The DCL-TBCs includes, (i) a nickel- or cobalt-based structural super-alloy substrate, (ii) a thin NiCrA1Y bonding coating (BC), (iii) a top ceramic layer (TC1) made from new ceramic materials mentioned above (e.g., LZ, La_2_Zr_2_O_7_) to provide effective thermal insulation and good sintering resistance, and (iv) an inside ceramic layer (TC2) made from the traditional ceramic material (YSZ, ZrO_2_-8%Y_2_O_3_) to reduce high level thermal expansion mismatch between TC1 and the combination of “BC+substrate” caused by low thermal expansion coefficient of TC1, as shown in Fig 1. Experiment results show that the DCL-TBCs have better thermal cycling performance than the traditional single YSZ layer TBCs [5–7, 9, 10].

It is well known that YSZ layer and LZ layer are usually fabricated by APS method, which means ZrO_2_-8%Y_2_O_3_ and La_2_Zr_2_O_7_ particles should be heated to their melting points (e.g. 2680°C for YSZ and 2300°C for LZ) [5] before they are deposited. Inevitably, a high level residual stress will generate in fabrication process of DCL-TBCs, due to the large Coefficients of Thermal Expansion (CTE) mismatches among the constituent layers [11–13]. It is considered as one of most important factors to affect micro cracks formations, propagations and failure in the as-sprayed DCL-TBCs, which has a significant value in evaluating the performance and service life of coating system [14, 15].

Indeed, the residual stress generation in coating/substrate system has attracted wide research interests in recent years, examples including the experiment observations [16, 17], Finite Element Method (FEM) simulations [18, 19] and theoretical models [20–23]. Among them, the theoretical model is especially suitable for predicting the trend of the residual stress generation of TBCs with different geometrical parameters and fabrication processes, therefore, in this work we focus on studying the residual stress generation in fabrication process of DCL-TBCs by using theoretical model. There are a lot of theoretical models have been established to estimate residual stress generation in coating/substrate system. One of the most famous method should be the Stoney’s equation, it is usually used to relate stress to curvature for thin coating/thick substrate system, where thickness of substrate is much bigger than that of coating, and residual stress in substrate can be negligible [20]; with increase of thicknesses ratio of coating to substrate, residual stress in substrate is no longer negligible, Timoshenko suggested calculating residual stress using beam theory [21]; after that, based on force and moment equilibrium, Freund, Suresh [22] and Zhang [23] proposed their theoretical models respectively to calculate residual stress generated by CTE mismatch in multi-layer coating system. However, DCL-TBCs as an advanced TBCs proposed in recent years, the residual stress generated in its whole fabrication process has rarely been studied by theoretical model. Therefore, a theoretical model which can well describe the residual stress generation in the fabrication process and consider the variation of thermal-mechanical properties of each materials is needed.

Meanwhile, as a double-layer ceramic coating system, the different layer thickness ratio of YSZ to LZ layers, which has already been approved to have a significant effect on durability and performance of DCL-TBCs, has been studied in previous works [5, 24–26]. Such as, Dai et al found that cycling live of DCL-TBCs is strongly dependent on the thickness ratio of LZ to YSZ by doing thermal cycling test [5], Han et al found the effect of the thickness ratio of LZ to YSZ greatly affects the heat insulation behavior of DCL-TBCs by using FEM [24], A. Moridi et al [25] studied how the YSZ thickness affects residual stress distribution under thermo-mechanical cyclic loading. However, studies on how this thickness ratio affects residual stress generation in the fabrication process of DCL-TBCs are relatively less, especially for the theoretical studies.

With thermal spray coatings being widely applied in industry, many new technologies have been employed to meet the improving requirements of thermal spray coatings. The pre-heating treatment, this treatment pre-heats the substrate to a given temperature level by using the radiation heating or the electron beam heating methods before the coating deposition process [27]. Previous works show that this treatment has a significant effect on the coating/substrate interface properties, residual stress distributions and thermal shock resistance ability [27–29]. As mentioned above, a high level residual stress will be generated in the fabrication of DCL-TBCs, therefore, it is of great significance to investigate the effect of pre-heating treatment on residual stress generation in the fabrication of DCL-TBCs.

In this work, based on the practical fabrication process of DCL-TBCs and the force and moment equilibrium, a theoretical model is developed at first, to predict residual stress generated in the whole fabrication process of DCL-TBCs, in which the temperature effects on material properties of DCL-TBCs were also incorporated. In addition, a FEM simulation has also been carried out to verify the developed theoretical model. Different thickness ratios of YSZ layer to LZ layers are discussed to reveal how it affects residual stress generation in fabrication process of DCL-TBCs. In addition, the effect of pre-heating treatment on residual stress generation are discussed as well.

### Statement of the Problem

**Fig 2:**
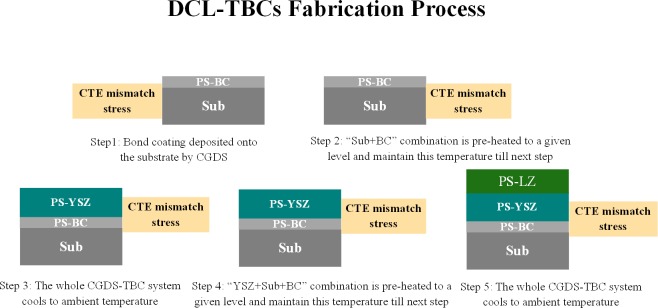
Schematic diagram of the whole fabrication process of DCL-TBCs.

One of the frequently-used fabrication process of DCL-TBCs is shown as follows: a nickel-based structural super-alloy substrate (e.g., INCONEL 617) is fixed at an atmospheric environment; after that, NiCrA1Y powder particles are heated to a specified temperature (e.g. 500°C) and deposited onto the surface of substrate to form a bond coating (BC) with a small thickness (e.g., 100μm); then, the “BC+substrate” combination cools down to the room temperature (e.g., 23°C); next, this “BC+substrate” combination will be pre-heated to a specified temperature (e.g., 500°C) and keeps this till the next action; YSZ powder particles are heated to melting temperature (e.g., 2680°C) and deposited onto the surface of bond coating layer by layer till the thickness of YSZ layer reachs a given thickness level (e.g., 150μm); subsequently, the “substrate + BC + YSZ” combination cools down to the room temperature; before LZ deposition process, the “substrate+BC+YSZ” combination will be pre-heated to a given temperature (e.g., 500°C) and keeps this till the next step; then, LZ powder particles deposited onto the top of YSZ layer with its melting temperature till the thickness reaches 150μm; at last, the whole DCL-TBCs will nature cooling down to room temperature, the fabrication diagram of DCL-TBCs is shown in Fig 2. The summation of residual stress generated in each step is the total or final residual stress in this paper.

During the whole fabrication process of DCL-TBCs, the total residual stress mainly comes from 5 sources:

1. In step-1: “bond coating deposition” process, residual stress arisen by CTE mismatch between BC and substrate;
2. In step-2: “BC+ substrate” combination pre-heating process, residual stress generated by CTE mismatch between BC and substrate;
3. In step-3: “YSZ+BC+substrate” combination cooling process, residual stress generated by CTE mismatch between BC, substrate and YSZ layer;
4. In step-4: “YSZ+BC+substrate” combination pre-heating process, residual stress generated by CTE mismatch between these 3 layers;
5. In step-5: “LZ+YSZ+BC+ substrate” combination natural cooling to room temperature process, residual stress generated by CTE mismatch between these 4 layers.

As mentioned above, all layers in DCL-TBCs are considered to be linearly elastic and isotropic, the current theoretical model is assumed under the equal-biaxial in-plane stress state (*σ*_*x*_ = *σ*_*z*_, and *σ*_*y*_ = 0). Effective Young’s modulus and Coefficients of Thermal Expansion of each layer of DCL-TBCs are ($E_{\text{Sub}}^{*}$,*α*_Sub_), ($E_{\text{BC}}^{*}$,*α*_BC_), ($E_{\text{YSZ}}^{*}$,*α*_YSZ_) and ($E_{\text{LZ}}^{*}$,*α*_LZ_) respectively, where the effective Young’s modulus can be obtained by $E_{}^{*}=\frac{E}{1-v}$. Stress release and plastic deformation mechanisms are not taken into amount in this work, therefore, these two hypothesizes may overrate residual stress arisen.

Since residual stresses generated in LZ layer and YSZ layer are essential for its dependability, one divides LZ layer and YSZ layer into a series of thin layers. The total thickness of ceramic layers is usually about 300μm [5, 24, 30]. To study the effect of thickness ratio of YSZ to LZ layers, a series of thickness ratios, i.e., YSZ:LZ = 5:1 (YSZ: 250μm, LZ: 50μm), YSZ:LZ = 4:2 (YSZ: 200μm, LZ: 100μm), YSZ:LZ = 3:3 (YSZ: 150μm, LZ: 150μm), YSZ:LZ = 2:4 (YSZ: 100μm, LZ: 200μm), and YSZ:LZ = 1:5 (YSZ: 50μm, LZ: 250μm) have been well discussed. The thickness of BC and substrate are 100μm and 1500μm [31, 32], respectively. In addition, according to works of Clyne [33] and Song [34], quenching stress have been approved to have a negligible effect on total residual stress in the fabrication of TBCs, therefore, residual stress generated by quenching stress in deposition processes of YSZ and LZ layers is not considered.

Residual stresses generated in the first 3 steps as shown in Fig 2 can be analyzed using the method developed in our previous work [34]. This part of work will focus on studying residual stress generated in step-4 and step-5.

### The pre-heating process of “YSZ+BC+substrate” combination

**Fig 3:**
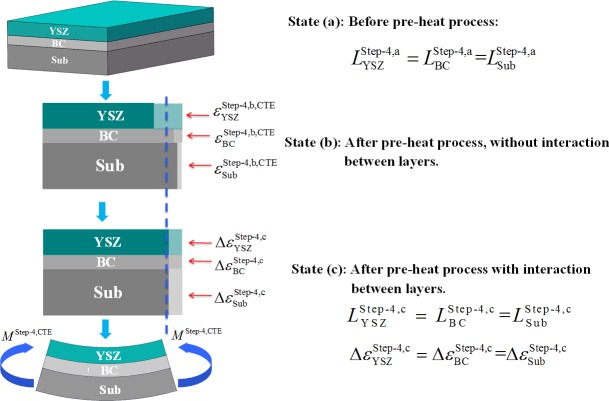
Schematic of the “YSZ+BC+substrate” combination thermal deformation during the pre-heating process. (A) This combination is preheated from room temperature to a given temperature (e.g. 500°C). (B) From state “a” to “c”: before the pre-heating process of the “YSZ+BC+substrate” combination, constraint-free thermal deformation without interaction between different layers, and the end of the pre-heating process of “YSZ+BC+substrate” combination.

Before the deposition of LZ layer, the “YSZ+BC+substrate” combination will be heated to a given temperature level (such as 500°C). Based on force and moment equilibrium [22, 23, 35, 36], residual stresses arisen by CTE mismatches between substrate, BC and YSZ layer can be calculated as shown in Fig 3.

Before the YSZ layer, BC and substrate are heated, their lengths are the same, i.e. $L_{\text{YSZ}}^{\text{Step-}4,\text{a}}=L_{\text{BC}}^{\text{Step-}4,\text{a}}=L_{\text{Sub}}^{\text{Step-}4,\text{a}}$, as shown in Fig 3. Then, this combination will be heated to a given temperature, a series of unconstrained thermal strains $\varepsilon_{\text{YSZ}}^{\text{Step-}4,\text{b},\text{CTE}}$, $\varepsilon_{\text{BC}}^{\text{Step-}4,\text{b},\text{CTE}}$ and $\varepsilon_{\text{Sub}}^{\text{Step-}4,\text{b},\text{CTE}}$, which generated in this temperature increasing process, can be calculated as follows.

$$\varepsilon_{\text{YSZ}}^{\text{Step-}4,b,\text{CTE}}=\int_{T_{\text{room}}}^{T_{\text{YSZ-CET}4}}\alpha_{\text{YSZ}}(T)dT$$ (1)

$$\varepsilon_{\text{BC}}^{\text{Step-}4,b,\text{CTE}}=\int_{T_{\text{room}}}^{T_{\text{BC-CET}4}}\alpha_{\text{BC}}(T)dT$$ (2)

$$\varepsilon_{\text{Sub}}^{\text{Step-}4,b,\text{CTE}}=\int_{T_{\text{room}}}^{T_{\text{Sub-CET}4}}\alpha_{\text{Sub}}(T)dT$$ (3)

The constraint of thick nickel-based substrate may result in a series of forces $F_{\text{YSZ}}^{\text{Step-}4}$, $F_{\text{BC}}^{\text{Step-}4}$ and $F_{\text{Sub}}^{\text{Step-}4}$ among YSZ layer, BC and thick nickel-based substrate, respectively. Meanwhile, these in-plane forces will lead to a moment *M*^Step-4,CTE^. After the pre-heating process, the final strains in YSZ layer, BC and thick nickel-based substrate ($\Delta \varepsilon_{\text{YSZ}}^{\text{Step-}4,c}$, $\Delta \varepsilon_{\text{BC}}^{\text{Step-}4,c}$ and $\Delta \varepsilon_{\text{Sub}}^{\text{Step-}4,c}$) can be calculated as follows:

$$\Delta \varepsilon_{\text{YSZ}}^{\text{Step-}4,\text{c}}=\int_{T_{\text{room}}}^{T_{\text{YSZ-CET}4}}\alpha_{\text{YSZ}}(T)dT+\frac{F_{\text{YSZ}}^{\text{Step-}4}}{bh_{\text{YSZ}}E_{\text{YSZ}}}$$ (4)

$$\Delta \varepsilon_{\text{BC}}^{\text{Step-}4,\text{c}}=\int_{T_{\text{room}}}^{T_{\text{BC-CET}4}}\alpha_{\text{BC}}(T)dT+\frac{F_{\text{BC}}^{\text{Step-}4}}{bh_{\text{BC}}E_{\text{BC}}}$$ (5)

$$\Delta \varepsilon_{\text{Sub}}^{\text{Step-}4,c}=\int_{T_{\text{room}}}^{T_{\text{Sub-CET}4}}\alpha_{\text{Sub}}(T)dT+\frac{F_{\text{Sub}}^{\text{Step-}4}}{bh_{\text{Sub}}E_{\text{Sub}}}$$ (6)

The curvature increase of the “YSZ+BC+substrate” in this process is Δ*κ*^Step-4^, the in-plane forces $F_{\text{YSZ}}^{\text{Step-}4}$ $F_{\text{BC}}^{\text{Step-}4}$ and $F_{\text{Sub}}^{\text{Step-}4}$ can be obtained as follows:

$$F_{\text{YSZ}}^{\text{Step-}4}=\left[\frac{E_{\text{YSZ}}^{*}(1+\varepsilon_{\text{YSZ}}^{\text{Step-}4,b,\text{CTE}})h_{\text{YSZ}}+E_{\text{BC}}^{*}(1+\varepsilon_{\text{BC}}^{\text{Step-}4,b,\text{CTE}})h_{\text{BC}}+E_{\text{Sub}}^{*}(1+\varepsilon_{\text{Sub}}^{\text{Step-}4,b,\text{CTE}})h_{\text{Sub}}}{E_{\text{YSZ}}^{*}h_{\text{YSZ}}+E_{\text{BC}}^{*}h_{\text{BC}}+E_{\text{Sub}}^{*}h_{\text{Sub}}}-1-\varepsilon_{\text{YSZ}}^{\text{Step-}4,b,\text{CTE}}\right]A_{\text{YSZ}}E_{\text{YSZ}}^{*}$$ (7)

$$F_{\text{BC}}^{\text{Step-}4}=\left[\frac{E_{\text{YSZ}}^{*}(1+\varepsilon_{\text{YSZ}}^{\text{Step-}4,b,\text{CTE}})h_{\text{YSZ}}+E_{\text{BC}}^{*}(1+\varepsilon_{\text{BC}}^{\text{Step-}4,b,\text{CTE}})h_{\text{BC}}+E_{\text{Sub}}^{*}(1+\varepsilon_{\text{Sub}}^{\text{Step-}4,b,\text{CTE}})h_{\text{Sub}}}{E_{\text{YSZ}}^{*}h_{\text{YSZ}}+E_{\text{BC}}^{*}h_{\text{BC}}+E_{\text{Sub}}^{*}h_{\text{Sub}}}-1-\varepsilon_{\text{BC}}^{\text{Step-}4,b,\text{CTE}}\right]A_{\text{BC}}E_{\text{BC}}^{*}$$ (8)

$$F_{\text{Sub}}^{\text{Step-}4}=\left[\frac{E_{\text{YSZ}}^{*}(1+\varepsilon_{\text{YSZ}}^{\text{Step-}4,b,\text{CTE}})h_{\text{YSZ}}+E_{\text{BC}}^{*}(1+\varepsilon_{\text{BC}}^{\text{Step-}4,b,\text{CTE}})h_{\text{BC}}+E_{\text{Sub}}^{*}(1+\varepsilon_{\text{Sub}}^{\text{Step-}4,b,\text{CTE}})h_{\text{Sub}}}{E_{\text{YSZ}}^{*}h_{\text{YSZ}}+E_{\text{BC}}^{*}h_{\text{BC}}+E_{\text{Sub}}^{*}h_{\text{Sub}}}-1-\varepsilon_{\text{Sub}}^{\text{Step-}4,b,\text{CTE}}\right]A_{\text{Sub}}E_{\text{Sub}}^{*}$$ (9)

Finally, residual stresses generated in YSZ and BC layers and substrate after this pre-heating process can be calculated as follows:

$$\begin{array}{l} \sigma_{\text{YSZ}|y}^{\text{Step-}4}=\left[\frac{E_{\text{YSZ}}^{*}(1+\varepsilon_{\text{YSZ}}^{\text{Step-}4,b,\text{CTE}})h_{\text{YSZ}}+E_{\text{BC}}^{*}(1+\varepsilon_{\text{BC}}^{\text{Step-}4,b,\text{CTE}})h_{\text{BC}}+E_{\text{Sub}}^{*}(1+\varepsilon_{\text{Sub}}^{\text{Step-}4,b,\text{CTE}})h_{\text{Sub}}}{E_{\text{YSZ}}^{*}h_{\text{YSZ}}+E_{\text{BC}}^{*}h_{\text{BC}}+E_{\text{Sub}}^{*}h_{\text{Sub}}}-1-\varepsilon_{\text{YSZ}}^{\text{Step-}4,b,\text{CTE}}\right]E_{\text{YSZ}}^{*}\\ \;+\Delta \kappa_{}^{\text{Step-}4}E_{\text{YSZ}}^{*}(y_{}-\delta_{}^{\text{Step-}4}) \end{array}$$ (10)

$$\begin{array}{l} \sigma_{\text{BC}|y}^{\text{Step-}4}=\left[\frac{E_{\text{YSZ}}^{*}(1+\varepsilon_{\text{YSZ}}^{\text{Step-}4,b,\text{CTE}})h_{\text{YSZ}}+E_{\text{BC}}^{*}(1+\varepsilon_{\text{BC}}^{\text{Step-}4,b,\text{CTE}})h_{\text{BC}}+E_{\text{Sub}}^{*}(1+\varepsilon_{\text{Sub}}^{\text{Step-}4,b,\text{CTE}})h_{\text{Sub}}}{E_{\text{YSZ}}^{*}h_{\text{YSZ}}+E_{\text{BC}}^{*}h_{\text{BC}}+E_{\text{Sub}}^{*}h_{\text{Sub}}}-1-\varepsilon_{\text{BC}}^{\text{Step-}4,b,\text{CTE}}\right]E_{\text{BC}}^{*}\\ \;+\Delta \kappa_{}^{\text{Step-}4}E_{\text{BC}}^{*}(y_{}-\delta_{}^{\text{Step-}4}) \end{array}$$ (11)

$$\begin{array}{l} \sigma_{\text{Sub}|y}^{\text{Step-}4}=\left[\frac{E_{\text{YSZ}}^{*}(1+\varepsilon_{\text{YSZ}}^{\text{Step-}4,b,\text{CTE}})h_{\text{YSZ}}+E_{\text{BC}}^{*}(1+\varepsilon_{\text{BC}}^{\text{Step-}4,b,\text{CTE}})h_{\text{BC}}+E_{\text{Sub}}^{*}(1+\varepsilon_{\text{Sub}}^{\text{Step-}4,b,\text{CTE}})h_{\text{Sub}}}{E_{\text{YSZ}}^{*}h_{\text{YSZ}}+E_{\text{BC}}^{*}h_{\text{BC}}+E_{\text{Sub}}^{*}h_{\text{Sub}}}-1-\varepsilon_{\text{Sub}}^{\text{Step-}4,b,\text{CTE}}\right]E_{\text{Sub}}^{*}\\ \;+\Delta \kappa_{}^{\text{Step-}4}E_{\text{Sub}}^{*}(y_{}-\delta_{}^{\text{Step-}4}) \end{array}$$ (12)

The neutral axis of “YSZ+BC+substrate” combination *δ*^Step-4^, the moment *M*^Step-4,CTE^, the bending stiffness *D*^Step-4^, and the curvature increase Δ*κ*^Step-4^ can be obtained as follows.

$$M_{}^{\text{Step-}4,\text{CTE}}=-\left[\begin{array}{l} F_{\text{YSZ}}^{\text{Step-}4}\left(H_{\text{BC}}+\frac{H_{\text{YSZ}}-H_{\text{BC}}}{2}-\delta_{}^{\text{Step-}4}\right)+F_{\text{BC}}^{\text{Step-}4}\left(H_{\text{Sub}}+\frac{H_{\text{BC}}-H_{\text{Sub}}}{2}-\delta_{}^{\text{Step-}4}\right)\\ +F_{\text{Sub}}^{\text{Step-}4}\left(\frac{H_{\text{Sub}}}{2}-\delta_{}^{\text{Step-}4}\right) \end{array}\right]$$ (13)

$$\delta_{}^{\text{Step-}4}=\frac{1}{2}\frac{E_{\text{YSZ}}^{*}(H_{\text{YSZ}}{}_{}^{2}-H_{\text{BC}}{}_{}^{2})+E_{\text{BC}}^{*}(H_{\text{BC}}{}_{}^{2}-H_{\text{Sub}}{}_{}^{2})+E_{\text{Sub}}^{*}(H_{\text{Sub}}{}_{}^{2}-0_{}^{2})}{E_{\text{YSZ}}^{*}(H_{\text{YSZ}}-H_{\text{BC}})+E_{\text{BC}}^{*}(H_{\text{BC}}-H_{\text{Sub}})+E_{\text{Sub}}^{*}(H_{\text{Sub}}-0)}$$ (14)

$$\begin{array}{l} D_{}^{\text{Step-}4}=\frac{b}{3}E_{\text{YSZ}}^{*}\left[{\left(H_{\text{YSZ}}-\delta_{}^{\text{Step-}4}\right)}_{}^{3}-{\left(H_{\text{BC}}-\delta_{}^{\text{Step-}4}\right)}_{}^{3}\right]+\frac{b}{3}E_{\text{BC}}^{*}\left[{\left(H_{\text{BC}}-\delta_{}^{\text{Step-}4}\right)}_{}^{3}-{\left(H_{\text{Sub}}-\delta_{}^{\text{Step-}4}\right)}_{}^{3}\right]\\ \;+\frac{b}{3}E_{\text{Sub}}^{*}\left[{\left(H_{\text{Sub}}-\delta_{}^{\text{Step-}4}\right)}_{}^{3}-{\left(0-\delta_{}^{\text{Step-}4}\right)}_{}^{3}\right] \end{array}$$ (15)

$$\Delta \kappa^{\text{Step-}4}=\frac{-\left[F_{\text{YSZ}}^{\text{Step-}4}\left(H_{\text{BC}}+\frac{H_{\text{YSZ}}-H_{\text{BC}}}{2}-\delta^{\text{Step-}4}\right)+F_{\text{BC}}^{\text{Step-}4}\left(H_{\text{Sub}}+\frac{H_{\text{BC}}-H_{\text{Sub}}}{2}-\delta^{\text{Step-}4}\right)+F_{\text{Sub}}^{\text{Step-}4}\left(\frac{H_{\text{Sub}}}{2}-\delta^{\text{Step-}4}\right)\right]}{\left[\begin{array}{l} \frac{b}{3}E_{\text{YSZ}}^{*}\left[{\left(H_{\text{YSZ}}-\delta^{\text{Stpe-}4}\right)}^{3}-{\left(H_{\text{BC}}-\delta^{\text{Stpe-}4}\right)}^{3}\right]+\frac{b}{3}E_{\text{BC}}^{*}\left[{\left(H_{\text{BC}}-\delta^{\text{Stpe-}4}\right)}^{3}-{\left(H_{\text{Sub}}-\delta^{\text{Stpe-}4}\right)}^{3}\right]\\ +\frac{b}{3}E_{\text{Sub}}^{*}\left[{\left(H_{\text{Sub}}-\delta^{\text{Stpe-}4}\right)}^{3}-{\left(0-\delta^{\text{Stpe-}4}\right)}^{3}\right] \end{array}\right]}$$ (16)

### The natural cooling process of DCL-TBCs

**Fig 4:**
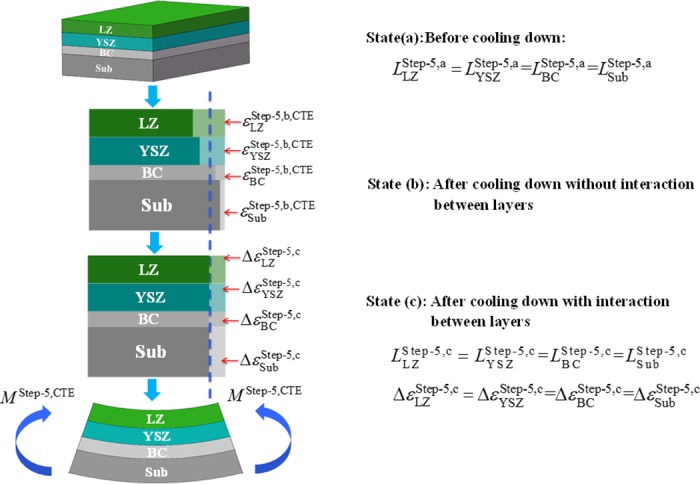
Schematic of “LZ+YSZ+BC+substrate” combination thermal deformation in the process of DCL-TBCs cools to room temperature. From state “a” to “c” (from top to bottom): the beginning of the “LZ+YSZ+BC+substrate” combination cooling down process, constraint-free thermal deformation, and the end of “LZ+YSZ+BC+substrate” combination cooling down process.

After LZ layer (La_2_Zr_2_O_7_) deposited onto the surface of YSZ layer, the whole DCL-TBCs naturally cools down to 23°C. Based on force and moment equilibrium, in this natural cooling process, residual stress arisen by CTE mismatches among the constituent layers of DCL-TBCs, can be obtained as shown in Fig 4.

At state “a”, the beginning state of the natural cooling process, lengths of constituent layers of DCL-TBCs are the same, i.e. $L_{\text{LZ}}^{\text{Step-}5,\text{a}}=L_{\text{YSZ}}^{\text{Step-}5,\text{a}}=L_{\text{BC}}^{\text{Step-}5,\text{a}}=L_{\text{Sub}}^{\text{Step-}5,\text{a}}$. Considering the coefficient of thermal expansions are temperature independent, the free thermal mismatch strains $\varepsilon_{\text{LZ}}^{\text{Step-}5,\text{b},\text{CTE}}$, $\varepsilon_{\text{YSZ}}^{\text{Step-}5,\text{b},\text{CTE}}$, $\varepsilon_{\text{BC}}^{\text{Step-}5,\text{b},\text{CTE}}$ and $\varepsilon_{\text{Sub}}^{\text{Step-}5,\text{b},\text{CTE}}$ can be obtained as follows:

$$\varepsilon_{\text{LZ}}^{\text{Step-}5,b,\text{CTE}}=\int_{T_{\text{LZ-CET}5}}^{T_{\text{room}}}\alpha_{\text{LZ}}(T)dT$$ (17)

$$\varepsilon_{\text{YSZ}}^{\text{Step-}5,b,\text{CTE}}=\int_{T_{\text{YSZ-CET}5}}^{T_{\text{room}}}\alpha_{\text{YSZ}}(T)dT$$ (18)

$$\varepsilon_{\text{BC}}^{\text{Step-}5,b,\text{CTE}}=\int_{T_{\text{BC-CET}5}}^{T_{\text{room}}}\alpha_{\text{BC}}(T)dT$$ (19)

$$\varepsilon_{\text{Sub}}^{\text{Step-}5,b,\text{CTE}}=\int_{T_{\text{Sub-CET}5}}^{T_{\text{room}}}\alpha_{\text{Sub}}(T)dT$$ (20)

Due to the constraint of substrate, forces $F_{\text{LZ}}^{\text{Step-}5}$, $F_{\text{YSZ}}^{\text{Step-}5}$, $F_{\text{BC}}^{\text{Step-}5}$ and $F_{\text{Sub}}^{\text{Step-}5}$ are arisen in constituent layers of DCL-TBCs. Meanwhile, those in-plane forces will lead to a moment *M*^Step-5,CTE^. The total strains in constituent layers of DCL-TBCs after the natural cooling process can be obtained as follows,

$$\Delta \varepsilon_{\text{LZ}}^{\text{Step-}5,c}=\int_{T_{\text{LZ-CET}5}}^{T_{\text{room}}}\alpha_{\text{LZ}}(T)dT+\frac{F_{\text{LC}}^{\text{Step-}5}}{bH_{\text{LZ}}E_{\text{LZ}}}$$ (21)

$$\Delta \varepsilon_{\text{YSZ}}^{\text{Step-}5,c}=\int_{T_{\text{YSZ-CET}5}}^{T_{\text{room}}}\alpha_{\text{YSZ}}(T)dT+\frac{F_{\text{YSZ}}^{\text{Step-}5}}{bH_{\text{YSZ}}E_{\text{YSZ}}}$$ (22)

$$\Delta \varepsilon_{\text{BC}}^{\text{Step-}5,c}=\int_{T_{\text{BC-CET}5}}^{T_{\text{room}}}\alpha_{\text{BC}}(T)dT+\frac{F_{\text{BC}}^{\text{Step-}5}}{bH_{\text{BC}}E_{\text{BC}}}$$ (23)

$$\Delta \varepsilon_{\text{Sub}}^{\text{Step-}5,c}=\int_{T_{\text{Sub-CET}5}}^{T_{\text{room}}}\alpha_{\text{Sub}}(T)dT+\frac{F_{\text{Sub}}^{\text{Step-}5}}{bH_{\text{Sub}}E_{\text{Sub}}}$$ (24)

The curvature increase of the DCL-TBCs Δ*κ*^Step-5^, and the in-plane forces $F_{\text{LZ}}^{\text{Step-}5}$, $F_{\text{YSZ}}^{\text{Step-}5}$, $F_{\text{BC}}^{\text{Step-}5}$ and $F_{\text{Sub}}^{\text{Step-}5}$ can be calculated as follows:

$$F_{\text{LZ}}^{\text{Step-}5}=\left[\frac{E_{\text{LZ}}^{*}(1+\varepsilon_{\text{LZ}}^{\text{Step-}5,b,\text{CTE}})h_{\text{LZ}}+E_{\text{YSZ}}^{*}(1+\varepsilon_{\text{YSZ}}^{\text{Step-}5,b,\text{CTE}})h_{\text{YSZ}}+E_{\text{BC}}^{*}(1+\varepsilon_{\text{BC}}^{\text{Step-}5,b,\text{CTE}})h_{\text{BC}}+E_{\text{Sub}}^{*}(1+\varepsilon_{\text{Sub}}^{\text{Step-}5,b,\text{CTE}})h_{\text{Sub}}}{E_{\text{LZ}}^{*}h_{\text{LZ}}+E_{\text{YSZ}}^{*}h_{\text{YSZ}}+E_{\text{BC}}^{*}h_{\text{BC}}+E_{\text{Sub}}^{*}h_{\text{Sub}}}-1-\varepsilon_{\text{LZ}}^{\text{Step-}5,b,\text{CTE}}\right]A_{\text{LZ}}E_{\text{LZ}}^{*}$$ (25)

$$F_{\text{YSZ}}^{\text{Step-}5}=\left[\frac{E_{\text{LZ}}^{*}(1+\varepsilon_{\text{LZ}}^{\text{Step-}5,b,\text{CTE}})h_{\text{LZ}}+E_{\text{YSZ}}^{*}(1+\varepsilon_{\text{YSZ}}^{\text{Step-}5,b,\text{CTE}})h_{\text{YSZ}}+E_{\text{BC}}^{*}(1+\varepsilon_{\text{BC}}^{\text{Step-}5,b,\text{CTE}})h_{\text{BC}}+E_{\text{Sub}}^{*}(1+\varepsilon_{\text{Sub}}^{\text{Step-}5,b,\text{CTE}})h_{\text{Sub}}}{E_{\text{LZ}}^{*}h_{\text{LZ}}+E_{\text{YSZ}}^{*}h_{\text{YSZ}}+E_{\text{BC}}^{*}h_{\text{BC}}+E_{\text{Sub}}^{*}h_{\text{Sub}}}-1-\varepsilon_{\text{YSZ}}^{\text{Step-}5,b,\text{CTE}}\right]A_{\text{YSZ}}E_{\text{YSZ}}^{*}$$ (26)

$$F_{\text{BC}}^{\text{Step-}5}=\left[\frac{E_{\text{LZ}}^{*}(1+\varepsilon_{\text{LZ}}^{\text{Step-}5,b,\text{CTE}})h_{\text{LZ}}+E_{\text{YSZ}}^{*}(1+\varepsilon_{\text{YSZ}}^{\text{Step-}5,b,\text{CTE}})h_{\text{YSZ}}+E_{\text{BC}}^{*}(1+\varepsilon_{\text{BC}}^{\text{Step-}5,b,\text{CTE}})h_{\text{BC}}+E_{\text{Sub}}^{*}(1+\varepsilon_{\text{Sub}}^{\text{Step-}5,b,\text{CTE}})h_{\text{Sub}}}{E_{\text{LZ}}^{*}h_{\text{LZ}}+E_{\text{YSZ}}^{*}h_{\text{YSZ}}+E_{\text{BC}}^{*}h_{\text{BC}}+E_{\text{Sub}}^{*}h_{\text{Sub}}}-1-\varepsilon_{\text{BC}}^{\text{Step-}5,b,\text{CTE}}\right]A_{\text{BC}}E_{\text{BC}}^{*}$$ (27)

$$F_{\text{Sub}}^{\text{Step-}5}=\left[\frac{E_{\text{LZ}}^{*}(1+\varepsilon_{\text{LZ}}^{\text{Step-}5,b,\text{CTE}})h_{\text{LZ}}+E_{\text{YSZ}}^{*}(1+\varepsilon_{\text{YSZ}}^{\text{Step-}5,b,\text{CTE}})h_{\text{YSZ}}+E_{\text{BC}}^{*}(1+\varepsilon_{\text{BC}}^{\text{Step-}5,b,\text{CTE}})h_{\text{BC}}+E_{\text{Sub}}^{*}(1+\varepsilon_{\text{Sub}}^{\text{Step-}5,b,\text{CTE}})h_{\text{Sub}}}{E_{\text{LZ}}^{*}h_{\text{LZ}}+E_{\text{YSZ}}^{*}h_{\text{YSZ}}+E_{\text{BC}}^{*}h_{\text{BC}}+E_{\text{Sub}}^{*}h_{\text{Sub}}}-1-\varepsilon_{\text{Sub}}^{\text{Step-}5,b,\text{CTE}}\right]A_{\text{Sub}}E_{\text{Sub}}^{*}$$ (28)

Therefore, in the cooling process, the residual stresses generated in constituent layers of DCL-TBCs can be obtained as follows:

$$\begin{array}{l} \sigma_{\text{LZ}|y}^{\text{Step-}5}=\left[\frac{E_{\text{LZ}}^{*}(1+\varepsilon_{\text{LZ}}^{\text{Step-}5,b,\text{CTE}})h_{\text{LZ}}+E_{\text{YSZ}}^{*}(1+\varepsilon_{\text{YSZ}}^{\text{Step-}5,b,\text{CTE}})h_{\text{YSZ}}+E_{\text{BC}}^{*}(1+\varepsilon_{\text{BC}}^{\text{Step-}5,b,\text{CTE}})h_{\text{BC}}+E_{\text{Sub}}^{*}(1+\varepsilon_{\text{Sub}}^{\text{Step-}5,b,\text{CTE}})h_{\text{Sub}}}{E_{\text{LZ}}^{*}h_{\text{LZ}}+E_{\text{YSZ}}^{*}h_{\text{YSZ}}+E_{\text{BC}}^{*}h_{\text{BC}}+E_{\text{Sub}}^{*}h_{\text{Sub}}}-1-\varepsilon_{\text{LZ}}^{\text{Step-}5,b,\text{CTE}}\right]E_{\text{LZ}}^{*}\\ \;+\Delta \kappa_{}^{\text{Step-}5}E_{\text{LZ}}^{*}(y_{}-\delta_{}^{\text{Step-}5}) \end{array}$$ (29)

$$\begin{array}{l} \sigma_{\text{YSZ}|y}^{\text{Step-}5}=\left[\frac{E_{\text{LZ}}^{*}(1+\varepsilon_{\text{LZ}}^{\text{Step-}5,b,\text{CTE}})h_{\text{LZ}}+E_{\text{YSZ}}^{*}(1+\varepsilon_{\text{YSZ}}^{\text{Step-}5,b,\text{CTE}})h_{\text{YSZ}}+E_{\text{BC}}^{*}(1+\varepsilon_{\text{BC}}^{\text{Step-}5,b,\text{CTE}})h_{\text{BC}}+E_{\text{Sub}}^{*}(1+\varepsilon_{\text{Sub}}^{\text{Step-}5,b,\text{CTE}})h_{\text{Sub}}}{E_{\text{LZ}}^{*}h_{\text{LZ}}+E_{\text{YSZ}}^{*}h_{\text{YSZ}}+E_{\text{BC}}^{*}h_{\text{BC}}+E_{\text{Sub}}^{*}h_{\text{Sub}}}-1-\varepsilon_{\text{YSZ}}^{\text{Step-}5,b,\text{CTE}}\right]E_{\text{YSZ}}^{*}\\ \;+\Delta \kappa_{}^{\text{Step-}5}E_{\text{YSZ}}^{*}(y_{}-\delta_{}^{\text{Step-}5}) \end{array}$$ (30)

$$\begin{array}{l} \sigma_{\text{BC}|y}^{\text{Step-}5}=\left[\frac{E_{\text{LZ}}^{*}(1+\varepsilon_{\text{LZ}}^{\text{Step-}5,b,\text{CTE}})h_{\text{LZ}}+E_{\text{YSZ}}^{*}(1+\varepsilon_{\text{YSZ}}^{\text{Step-}5,b,\text{CTE}})h_{\text{YSZ}}+E_{\text{BC}}^{*}(1+\varepsilon_{\text{BC}}^{\text{Step-}5,b,\text{CTE}})h_{\text{BC}}+E_{\text{Sub}}^{*}(1+\varepsilon_{\text{Sub}}^{\text{Step-}5,b,\text{CTE}})h_{\text{Sub}}}{E_{\text{LZ}}^{*}h_{\text{LZ}}+E_{\text{YSZ}}^{*}h_{\text{YSZ}}+E_{\text{BC}}^{*}h_{\text{BC}}+E_{\text{Sub}}^{*}h_{\text{Sub}}}-1-\varepsilon_{\text{BC}}^{\text{Step-}5,b,\text{CTE}}\right]E_{\text{BC}}^{*}\\ \;+\Delta \kappa_{}^{\text{Step-}5}E_{\text{BC}}^{*}(y_{}-\delta_{}^{\text{Step-}5}) \end{array}$$ (31)

$$\begin{array}{l} \sigma_{\text{Sub}|y}^{\text{Step-}5}=\left[\frac{E_{\text{LZ}}^{*}(1+\varepsilon_{\text{LZ}}^{\text{Step-}5,b,\text{CTE}})h_{\text{LZ}}+E_{\text{YSZ}}^{*}(1+\varepsilon_{\text{YSZ}}^{\text{Step-}5,b,\text{CTE}})h_{\text{YSZ}}+E_{\text{BC}}^{*}(1+\varepsilon_{\text{BC}}^{\text{Step-}5,b,\text{CTE}})h_{\text{BC}}+E_{\text{Sub}}^{*}(1+\varepsilon_{\text{Sub}}^{\text{Step-}5,b,\text{CTE}})h_{\text{Sub}}}{E_{\text{LZ}}^{*}h_{\text{LZ}}+E_{\text{YSZ}}^{*}h_{\text{YSZ}}+E_{\text{BC}}^{*}h_{\text{BC}}+E_{\text{Sub}}^{*}h_{\text{Sub}}}-1-\varepsilon_{\text{Sub}}^{\text{Step-}5,b,\text{CTE}}\right]E_{\text{Sub}}^{*}\\ \;+\Delta \kappa_{}^{\text{Step-}5}E_{\text{Sub}}^{*}(y_{}-\delta_{}^{\text{Step-}5}) \end{array}$$ (32)

The neutral axis of DCL-TBCs *δ*^Step-5^, the bending stiffness *D*^Step-5^, the moment *M*^Step-5,CTE^, and the curvature increase in step-5 Δ*κ*^Step-5^ can be obtained as follows:

$$M_{}^{\text{Step-}5,\text{CTE}}=-\left[\begin{array}{l} F_{\text{LZ}}^{\text{Step-}5}\left(H_{\text{YSZ}}+\frac{H_{\text{LZ}}-H_{\text{YSZ}}}{2}-\delta_{}^{\text{Step-}5}\right)+F_{\text{YSZ}}^{\text{Step-}5}\left(H_{\text{BC}}+\frac{H_{\text{YSZ}}-H_{\text{BC}}}{2}-\delta_{}^{\text{Step-}5}\right)\\ +F_{\text{BC}}^{\text{Step-}5}\left(H_{\text{Sub}}+\frac{H_{\text{BC}}-H_{\text{Sub}}}{2}-\delta_{}^{\text{Step-}5}\right)+F_{\text{Sub}}^{\text{Step-}5}\left(\frac{H_{\text{Sub}}}{2}-\delta_{}^{\text{Step-}5}\right) \end{array}\right]$$ (33)

$$\delta^{\text{Step-}5}=\frac{1}{2}\frac{E_{\text{LZ}}^{*}\left(H_{\text{LZ}}{}^{2}-H_{\text{YSZ}}{}^{2}\right)+E_{\text{YSZ}}^{*}\left(H_{\text{YSZ}}{}^{2}-H_{\text{BC}}{}^{2}\right)+E_{\text{BC}}^{*}\left(H_{\text{BC}}{}^{2}-H_{\text{Sub}}{}^{2}\right)+E_{\text{Sub}}^{*}\left(H_{\text{Sub}}{}^{2}-0^{2}\right)}{E_{\text{LZ}}^{*}\left(H_{\text{LZ}}-H_{\text{YSZ}}\right)+E_{\text{YSZ}}^{*}\left(H_{\text{YSZ}}-H_{\text{BC}}\right)+E_{\text{BC}}^{*}\left(H_{\text{BC}}-H_{\text{Sub}}\right)+E_{\text{Sub}}^{*}\left(H_{\text{Sub}}-0\right)}$$ (34)

$$\begin{array}{l} D_{}^{\text{Step-}5}=\frac{b}{3}E_{\text{LZ}}^{*}\left[{\left(H_{\text{LZ}}-\delta_{}^{\text{Step-}5}\right)}_{}^{3}-{\left(H_{\text{YSZ}}-\delta_{}^{\text{Step-}5}\right)}_{}^{3}\right]+\frac{b}{3}E_{\text{YSZ}}^{*}\left[{\left(H_{\text{YSZ}}-\delta_{}^{\text{Step-}5}\right)}_{}^{3}-{\left(H_{\text{BC}}-\delta_{}^{\text{Step-}5}\right)}_{}^{3}\right]\\ \;+\frac{b}{3}E_{\text{BC}}^{*}\left[{\left(H_{\text{BC}}-\delta_{}^{\text{Step-}5}\right)}_{}^{3}-{\left(H_{\text{Sub}}-\delta_{}^{\text{Step-}5}\right)}_{}^{3}\right]+\frac{b}{3}E_{\text{Sub}}^{*}\left[{\left(H_{\text{Sub}}-\delta_{}^{\text{Step-}5}\right)}_{}^{3}-{\left(0-\delta_{}^{\text{Step-}5}\right)}_{}^{3}\right] \end{array}$$ (35)

$$\begin{array}{l} \Delta \kappa^{\text{Step-}5}=\frac{-\left[\begin{array}{l} F_{\text{LZ}}^{\text{Step-}5}\left(H_{\text{YSZ}}+\frac{H_{\text{LZ}}-H_{\text{YSZ}}}{2}-\delta^{\text{Step-}5}\right)+F_{\text{YSZ}}^{\text{Step-}5}\left(H_{\text{BC}}+\frac{H_{\text{YSZ}}-H_{\text{BC}}}{2}-\delta^{\text{Step-}5}\right)\\ +F_{\text{BC}}^{\text{Step-}5}\left(H_{\text{Sub}}+\frac{H_{\text{BC}}-H_{\text{Sub}}}{2}-\delta^{\text{Step-}5}\right)+F_{\text{Sub}}^{\text{Step-}5}\left(\frac{H_{\text{Sub}}}{2}-\delta^{\text{Step-}5}\right) \end{array}\right]}{\frac{b}{3}E_{\text{LZ}}^{*}\left[{\left(H_{\text{LZ}}-\delta^{\text{Stpe-}5}\right)}^{3}-{\left(H_{\text{YSZ}}-\delta^{\text{Stpe-}5}\right)}^{3}\right]+\frac{b}{3}E_{\text{YSZ}}^{*}\left[{\left(H_{\text{YSZ}}-\delta^{\text{Stpe-}5}\right)}^{3}-{\left(H_{\text{BC}}-\delta^{\text{Stpe-}5}\right)}^{3}\right]}\\ \;+\frac{b}{3}E_{\text{BC}}^{*}\left[{\left(H_{\text{BC}}-\delta^{\text{Stpe-}5}\right)}^{3}-{\left(H_{\text{Sub}}-\delta^{\text{Stpe-}5}\right)}^{3}\right]+\frac{b}{3}E_{\text{Sub}}^{*}\left[{\left(H_{\text{Sub}}-\delta^{\text{Stpe-}5}\right)}^{3}-{\left(0-\delta^{\text{Stpe-}5}\right)}^{3}\right] \end{array}$$ (36)

### Total residual stress arisen in the fabrication process of DCL-TBCs

After the fabrication process, the total residual stress arisen in DCL-TBCs can be calculated by adding residual stresses arisen in these 5 steps together. A Matlab algorithm is carried out to estimate residual stresses generated in each step and the total residual stress at the end of the fabrication process. To study the effect of the thickness ratio of YSZ to LZ layers on residual stress generation, a series of different thickness ratio (i.e. LZ: 50μm, YSZ: 250μm; LZ: 100μm, YSZ: 200μm; LZ: 150μm, YSZ: 150μm; LZ: 200μm, YSZ: 100μm; LZ: 250μm, YSZ: 50μm) have been well discussed. In addition, to observe effect of some spray factors, such as the pre-heating treatment, different pre-heating temperatures (i.e. 23°C, 250°C, 500°C, 1000°C) have also been studied. Thermal-mechanical properties of DCL-TBCs are shown in Table 1 [37, 38].

**Table 1:** Physical properties of DCL-TBCs [39, 38].

	T(°C)	E(GPa)	K(W/m C)	C(J/kg C)	ρ (kg/m^3^)	α x 10^−6^(C^-1^)	υ
LZ	3	175	0.81	219	4810	4.5	0.12
	400	167	0.78	455		9.85	
	800	150	0.74	475			
	1200	135	0.77	515		10.17	
YSZ	25	17.5	1.05	483	5650	9.68	0.2
	400	-				-	
	800	-				9.88	
	1000	12.4				10.34	
BC	25	183	4.3	501	7320	-	0.3
	400	152	6.4	592		12.5	
	800	109	10.2	781		14.3	
	1000	-	16.1	764		16	
Ni-based alloy	25	211	11.5	431	8220	12.6	0.3
	400	188	17.3	524		14	
	800	157	23.8	627		15.4	
	1000	139	-	-		16.3	

### Numerical analysis

**Fig 5:**
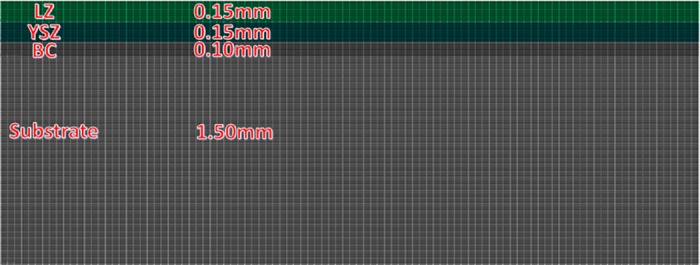
Mesh of FEM simulation.

Herein, finite element numerical analysis is carried out to verify the residual stress obtained by using the current theoretical model, and the commercial ABAQUS code is employed. In order to describe the in-plane stress state, two dimensional linear in-plane stress element CPS4R is employed for the whole DCL-TBCs, effective Young’s modulus $E_{}^{*}=\frac{E}{1-v}$ is used to describe equal-biaxial state. Elements in LZ layer, YSZ layer and BC are refined, the size of the elements in thickness direction and length direction are 0.01mm and 0.01mm, total elements are 70000, as shown in Fig 5. The mesh sensitivity is checked before calculations. The constitutive law of DCL-TBCs is assumed to be linear elasticity. In addition, thermal-mechanical properties of DCL-TBCs are the same as used in theoretical model, which is temperature dependent. The large deformation is employed to describe deformations generated in the whole fabrication process of DCL-TBCs.

## Results and Discussion

### Residual stress arisen in each step of fabrication process of DCL-TBCs

**Fig 6:**
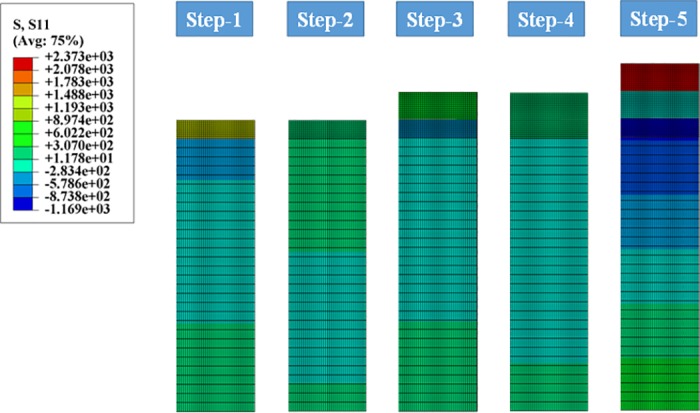
Residual stress generated in step 1~5 by FEM simulation.

The fabrication process of DCL-TBCs is constituted by 5 steps, residual stress distribution throughout DCL-TBCs varies during the whole fabrication process, therefore, studying residual stress generation in each step will help to forecast crack nucleation, generation and propagation inside DCL-TBCs, which has a significant value in optimizing the fabrication process and improving the quantities of DCL-TBCs. To study residual stresses arisen in each step in the fabrication process of DCL-TBCs, reference DCL-TBCs parameters have been chosen, i.e. the thicknesses of LZ layer, YSZ layer, BC and substrate are 150μm, 150μm, 100μm and 1500μm, respectively. In step-1, BC cools down to room temperature from 550°C (Temperature of BC particle during “BC deposition process”), in step-2, “BC+substrate” combination is pre-heated to 500°C, in step-3, the combination of “substrate (500°C)+BC (500°C)+YSZ layer (2680°C)” cools down to room temperature (23°C), in step-4, “YSZ+BC+substrate” combination is pre-heated to 500°C, in step-5, the whole DCL-TBCs (substrate (500°C)+BC (500°C)+YSZ layer (500°C)+LZ layer (2300°C)) cools down to room temperature. Meanwhile, a FEM simulation has been carried out to verify our theoretical model, and the results are shown in Fig 6.

**Fig 7:**
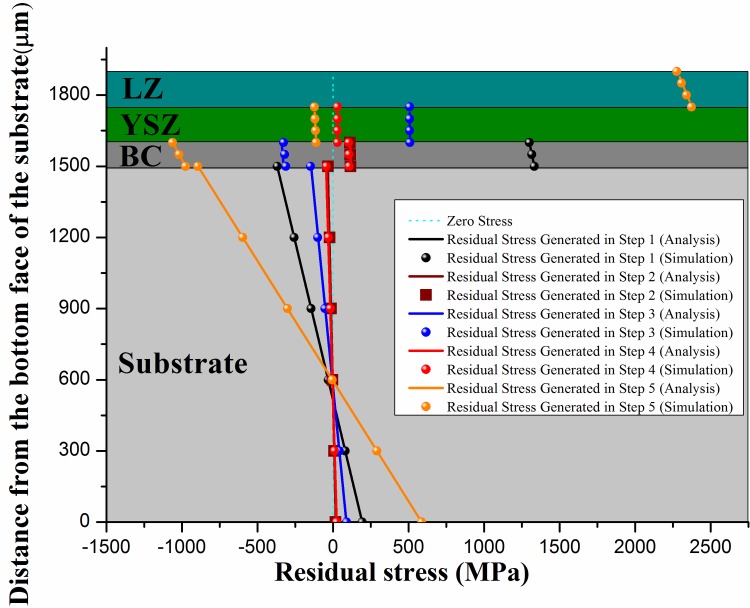
Comparison of residual stress generated during step 1~5 by theoretical model and FEM.

Residual stresses arisen in each step of the whole fabrication process of DCL-TBCs by theoretical model are shown in Fig 7. Meanwhile, a series of discrete FEM simulation results are also displayed in Fig 7 to verify our theoretical model, both results show good agreement with each other, as shown in Fig 7.

According to the results shown in Fig 7, one finds that: (i) In step-1, “bond coating deposition” process, BC cools down to room temperature (e.g., 23°C) from 550°C. Meanwhile, a great shrinkage occurs in BC owing to the significant CTE mismatch between BC and substrate. Residual stress arisen in this step in BC is therefore tensile with a notable magnitude. (ii) Residual stress arisen in step-2 contributes little to the total residual stress and it could be attributed to the similarity of thermal-mechanical properties of substrate and BC, as shown in Table 1. On the other hand, BC and substrate are heated to the given temperature and the CTE mismatch between BC and substrate is small. (iii) Residual stress generated in YSZ layer mainly results from step-3. In this step, YSZ layer cools down to room temperature (e.g., 23°C) from the deposition temperature of YSZ particles (e.g., 2680°C). The temperature variation in YSZ layer is nearly 5.5 times bigger than that in BC and substrate, which leads to a large tensile residual stress in YSZ layer. (iv). The combination of “YSZ+BC+ substrate” is preheated to a same temperature. Besides, the CTE differences of each layer in “YSZ+BC+ substrate” are very small. As found in step-2, residual stress generated in step-4 contributes little to the total residual stress. (v) Residual stress in LZ layer is only contributed by step-5. In step-5, the whole DCL-TBCs, LZ layer, YSZ layer, BC and substrate cools down to room temperature from 2300°C (deposition temperature of LZ particles), 500°C, 500°C and 500°C respectively. The temperature change in LZ layer is almost 5 times bigger than that in YSZ layer, BC and substrate. Moreover, the magnitude of elasticity modulus of LZ layer is big (Table 1). Thus significant tensile residual stress is generated in LZ layer in this step.

Residual stresses generated in step-2 (as shown in (ii)) and step-4 (as shown in (iv)) have little contributions to total residual stress, but this doesn’t mean pre-heating treatment just has small effect on total residual stress, on the contrary, pre-heating treatment has a significant effect on residual stress generated in DCL-TBCs, especially in LZ layer, which will be shown in following section (Effect of pre-heating treatment).

### Final/Total residual stress

**Fig 8:**
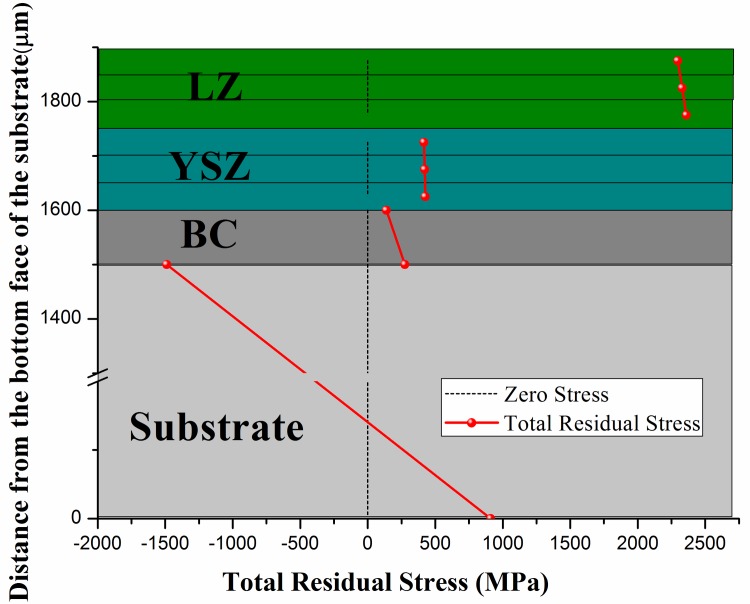
Final/Total residual stress generated in the whole fabrication process of DCL-TBCs.

Considering that all layers in DCL-TBCs are assumed to be isotropic, have linear elasticity, final/total residual stress arisen in the fabrication process of DCL-TBCs can be obtained by adding residual stresses generated in all 5 steps mentioned above together, result can be found in Fig 8.

By analyzing results in Fig 8, there are: (i) Residual stress in LZ layer is tensile stress, and the tensile stress is becoming smaller from the bottom to the top of LZ layer. That’s because the whole DCL-TBCs bends towards LZ layer after the fabrication process, the neutral axis is in the substrate, thus, bending stress in LZ layer is compressive stress and with the increasing of distance between the location inside LZ layer and the neutral axis, this compressive stress is increasing, too. (ii) Final residual stress in YSZ layer is tensile stress, as shown in section 3.1, residual stress in YSZ layer is contributed by 3 parts, tensile stress generated in step-3 and step-4, compressive stress generated in step-5, respectively, since the magnitude of tensile stress generated in step-3 is nearly 5 times bigger than that of compressive stress generated in step-5, so the final residual stress in YSZ layer is tensile stress, which also shows residual stress in YSZ layer is mainly contributed in step-3. (iii) Final residual stress in BC is also tensile stress, there are obvious stress difference in YSZ layer/BC interface and BC/substrate interface, this adjust well to BC’s principle function that BC deposited between YSZ layer and BC is to coordinate in YSZ layer and substrate [40]. (iv) There is an obvious stress drop in the BC/substrate interface. (v) The residual stress in Substrate is compressive stress at the top of substrate, and becoming to be tensile stress at the bottom of substrate, as mentioned above, the whole DCL-TBCs bends toward LZ layer after the fabrication process, bending stress is compressive stress at the top, the magnitude of compressive stress is increasing when approaching the top surface of substrate, meanwhile, bending stress is tensile stress at the bottom, and the magnitude of tensile stress is increasing when approaching the bottom of substrate.

### Effect of pre-heating treatment

**Fig 9:**
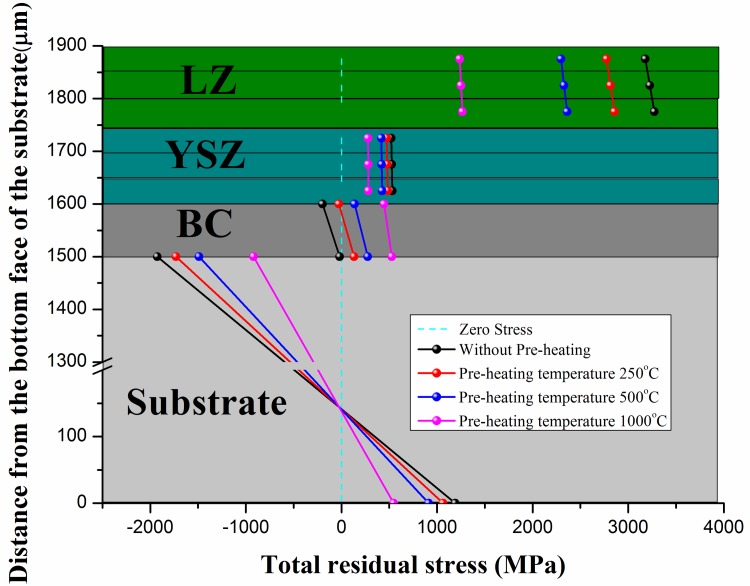
Comparison of final residual stress generated in the fabrication of DCL-TBCs with different pre-heating temperatures. Different pre-heating temperatures are: i.e. 23°C, 250°C, 500°C, 1000°C.

The pre-heating treatment is an effective way to improve residual stress arisen in the fabrication process of DCL-TBCs. In this work, in step-2 and step-4, the combinations of “BC+substrate” and “YSZ+BC+substrate” are pre-heated to 500°C before the deposition process of YSZ layer and LZ layer. To investigate the effect of pre-heating treatment, a set of specified temperatures (i.e. 23°C, 250°C, 500°C, 1000°C) has been studied in this part. Results are shown in Fig 9.

Inspection of results shown in Fig 9 reveals that: (i) Increasing pre-heating temperature can obviously decrease the magnitude of residual stresses in LZ layer, YSZ layer and substrate. Meanwhile, it can increase the magnitude of residual stress in BC. (ii) Pre-heating treatment can significantly decrease residual stress in LZ layer. The average residual stress in LZ layer decreases from 3226MPa (without pre-heating treatment) to 1251MPa (1000°C pre-heating temperature). (iii). Pre-heating treatment can reduce residual stress in YSZ layer. Although the magnitude of residual stress in YSZ layer doesn’t decrease significantly, this treatment is still helpful to improve the durability of YSZ layer. (iv) Increasing pre-heating temperature may increase residual stress in BC. However, considering the fact that BC is constituted by metal material, increase of residual stress in BC has no negative effect on reliability of BC. (v) Increasing pre-heating temperature will decrease residual stress arisen in substrate. Therefore, pre-heating treatment is widely employed in engineering industry.

### Effect of thickness ratio of YSZ to LZ layers

**Fig 10:**
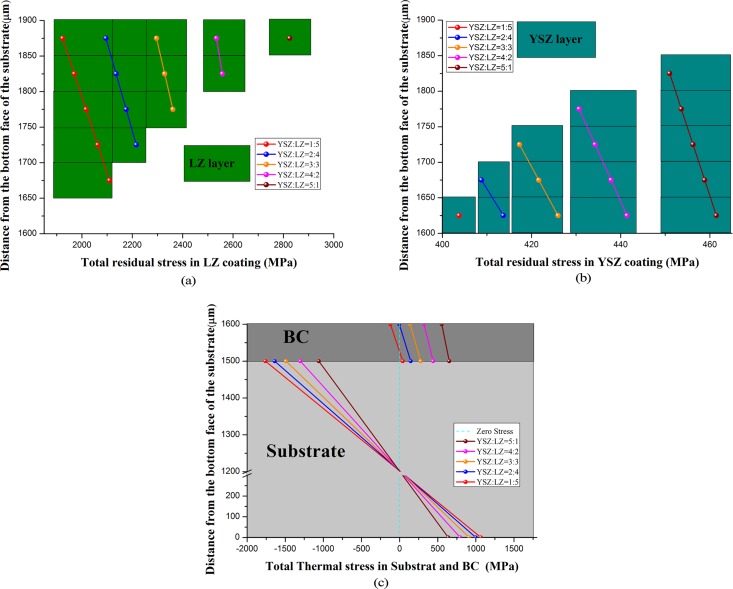
Comparison of final residual stress generated with different thickness ratios of YSZ to LZ layers. (A) Different thickness ratios of YSZ to LZ layers are: i.e. YSZ: 250μm, LZ: 50μm; YSZ: 200μm, LZ: 100μm; YSZ: 150μm, LZ: 150μm; YSZ: 100μm, LZ: 200μm; and YSZ: 50μm, LZ: 250μm. (B) (a-c) respect residual stress generated in LZ layer, YSZ layer and the combination of “substrate + BC”, respectively.

As shown in Cao and Dai’s work [5], the thickness ratio of YSZ layer to LZ layer has a significant effect on the cycling lives of DCL-TBCs. In order to investigate the effect of thickness ratio on the residual stress generation in the fabrication process of DCL-TBCs, in this work, a series of different thickness ratios of YSZ to LZ layers, (i.e., YSZ: 250μm, LZ: 50μm; YSZ: 200μm, LZ: 100μm; YSZ: 150μm, LZ: 150μm; YSZ: 100μm, LZ: 200μm; and YSZ: 50μm, LZ: 250μm) have been studied, residual stresses generated in each layer are shown in Fig 10 (A), 10(B) and 10(C), respectively.

According to the results shown in Fig 10, one finds that: (i) With the increase of thickness ratio of YSZ layer to LZ layer, magnitudes of residual stresses in LZ layer, YSZ layer and BC will be improved. With the increase of thickness ratio of YSZ to LZ layers, the stiffness of the combination “LZ+YSZ+BC” will be decreased (The Young modulus of LZ layer is much bigger than that of YSZ layer, see Table 1), thus, the constraint effect of substrate to confine the free shrinkage of the combination “LZ+YSZ+BC” is decreasing, therefore, their relative tensile strain are decreasing, so tensile stresses in LZ layer, YSZ layer and BC are decreasing. (ii) For each DCL-TBCs with a specific thickness ratio of YSZ to LZ layers, the magnitudes of residual stresses in LZ layer, YSZ layer and BC decrease from their top surface to the bottom, respectively, as shown in Fig 10(A) and 10(B). Since the whole DCL-TBCs bends towards LZ layer and the neutral axis is in the substrate, thus, bending stress in LZ layer, YSZ layer and BC are compressive stress, and this compressive stress is increasing from the bottom to the top of each layer. (iii) With the decrease of thickness ratio of YSZ to LZ, the magnitudes of residual stresses in substrate is increasing.

Although residual stresses in LZ and YSZ layers can be decreased by decreasing the thickness ratio of YSZ to LZ layers as shown in Fig 10(A) and 10(B), fracture toughness of LZ layer is smaller than that of YSZ layer. Therefore, increase of thickness of LZ layer may lead to more cracks in DCL-TBCs. This may result in the fracture propagation and significantly reduce the service life of DCL-TBCs. Optimum design of DCL-TBCs, shall synthetically consider the magnitude of residual stress, fracture toughness, thermal insulation properties, etc.

## Conclusions

In this work, a theoretical model was developed to estimate residual stress arisen in the fabrication process of DCL-TBCs. The effects of thickness ratio of YSZ layer to LZ layer and pre-heating treatment on residual stress were discussed. A FEM simulation was also performed to validate the method presented in this study. The main conclusions can be drawn as:

1. The final residual stress mainly originates from the processes of bond coating deposition (step-1), “YSZ+BC+substrate” and the whole DCL-TBCs cooling down to ambient temperature processes (step-3 and step-5). Residual stresses generated in the processes of “BC+substrate” pre-heating (step-2) and “YSZ+BC+substrate” pre-heating (step-4) have a little contribution to the final residual stress.
2. Final residual stresses in LZ and YSZ layers are tensile with remarkable magnitudes. BC layer is also subjected to tensile residual stress. The residual stress in substrate is compressive at the top of substrate, and it becomes tensile at the bottom of substrate. Meanwhile, there are significant stress drops at the LZ/YSZ and BC layer/substrate interfaces.
3. Increasing the pre-heating temperature can obviously decrease the magnitude of residual stresses in LZ layer, YSZ layer and substrate, but increase the magnitude of residual stress in BC.
4. With the increase of the thickness ratio of YSZ to LZ layers, magnitudes of residual stresses arisen in LZ layer and YSZ layer will increase while residual stress in substrate will decrease.

## Supporting Information

S1 File Data underlying findings. (XLSX)
